# Racial-Ethnic Inequity in Young Adults With Type 1 Diabetes

**DOI:** 10.1210/clinem/dgaa236

**Published:** 2020-05-08

**Authors:** Shivani Agarwal, Lauren G Kanapka, Jennifer K Raymond, Ashby Walker, Andrea Gerard-Gonzalez, Davida Kruger, Maria J Redondo, Michael R Rickels, Viral N Shah, Ashley Butler, Jeffrey Gonzalez, Alandra S Verdejo, Robin L Gal, Steven Willi, Judith A Long

**Affiliations:** 1 Fleischer Institute for Diabetes and Metabolism, New York-Regional Center for Diabetes Translational Research, Division of Endocrinology, Albert Einstein College of Medicine, Bronx, NY; 2 Jaeb Center for Health Research, Tampa, FL; 3 Children’s Hospital Los Angeles, Los Angeles, CA; 4 University of Florida, Gainesville, FL; 5 Barbara Davis Center for Diabetes, University of Colorado Anschutz Medical Campus, Aurora, CO; 6 Henry Ford Medical Center, Detroit, MI; 7 Baylor College of Medicine, Texas Children’s Hospital, Houston, TX; 8 Institute for Diabetes, Obesity & Metabolism, University of Pennsylvania Perelman School of Medicine, Philadelphia, PA; 9 Ferkauf Graduate School of Psychology, Yeshiva University, Bronx, NY; 10 Children’s Hospital of Philadelphia, Philadelphia, PA; 11 Corporal Michael J. Crescenz VA Medical Center, Philadelphia, PA

**Keywords:** type 1 diabetes, young adults, healthcare disparities, inequity, social determinants of health

## Abstract

**Context:**

Minority young adults (YA) currently represent the largest growing population with type 1 diabetes (T1D) and experience very poor outcomes. Modifiable drivers of disparities need to be identified, but are not well-studied.

**Objective:**

To describe racial-ethnic disparities among YA with T1D and identify drivers of glycemic disparity other than socioeconomic status (SES).

**Design:**

Cross-sectional multicenter collection of patient and chart-reported variables, including SES, social determinants of health, and diabetes-specific factors, with comparison between non-Hispanic White, non-Hispanic Black, and Hispanic YA and multilevel modeling to identify variables that account for glycemic disparity apart from SES.

**Setting:**

Six diabetes centers across the United States.

**Participants:**

A total of 300 YA with T1D (18-28 years: 33% non-Hispanic White, 32% non-Hispanic Black, and 34% Hispanic).

**Main Outcome:**

Racial-ethnic disparity in HbA1c levels.

**Results:**

Non-Hispanic Black and Hispanic YA had lower SES, higher HbA1c levels, and much lower diabetes technology use than non-Hispanic White YA (*P* < 0.001). Non-Hispanic Black YA differed from Hispanic, reporting higher diabetes distress and lower self-management (*P* < 0.001). After accounting for SES, differences in HbA1c levels disappeared between non-Hispanic White and Hispanic YA, whereas they remained for non-Hispanic Black YA (+ 2.26% [24 mmol/mol], *P* < 0.001). Diabetes technology use, diabetes distress, and disease self-management accounted for a significant portion of the remaining non-Hispanic Black–White glycemic disparity.

**Conclusion:**

This study demonstrated large racial-ethnic inequity in YA with T1D, especially among non-Hispanic Black participants. Our findings reveal key opportunities for clinicians to potentially mitigate glycemic disparity in minority YA by promoting diabetes technology use, connecting with social programs, and tailoring support for disease self-management and diabetes distress to account for social contextual factors.

Incidence of type 1 diabetes is increasing in the United States, especially among Hispanic and non-Hispanic Black (Black) young adults (YA), ages 18 to 25 years (1-3). Minority YA groups exhibit some of the highest HbA1c levels, rates of hospitalizations, psychiatric comorbidity, and mortality among all age groups with type 1 diabetes, highlighting major racial-ethnic disparity in outcomes (4-9). Nevertheless, minority YA with type 1 diabetes are not well-studied, and drivers of disparities other than socioeconomic status (SES) are not clearly defined. Moreover, it is unknown whether there are potentially important differences between Black and Hispanic groups that could allow for tailoring of interventions.

Young adults are at a critical inflection point of newfound independence both in life and in disease self-management (10). As a result of competing financial, social, educational, and geographic shifts, YA infrequently interface with health care and may miss key interventional opportunities to prevent complications in adulthood (5, 11, 12). In general, YA experience deleterious short- and long-term outcomes (13-17). Minority status adds to this increased baseline risk. Prior literature notes that biological differences in glycation of hemoglobin between Black and non-Hispanic White (White) groups may account for apparent disparities in glycemic control. However, the estimated contribution of biological differences to glycemic control only accounts for a small proportion of the observed disparities between racial-ethnic minority and White groups (18). Thus, disparities may largely stem from social contextual factors, but this remains understudied.

The Social Determinants of Health (SDOH) model describes a set of factors that affect the health of individuals and communities beyond personal choices that are a product of their life experiences, physical, social, and economic environments (19, 20). Social determinants are multilevel and include factors such as individual and neighborhood poverty level, literacy, educational attainment, food security, and adverse childhood experiences, among others. Although it is well-established that SDOH greatly influence health outcomes in minority and low SES populations (19, 21), several gaps remain in the literature: (1) studies of YA minority populations with type 1 diabetes are scarce and could show different results compared with adults or youth given their unique developmental needs; (2) existing research does not examine the effect of multiple coincident SDOH and other disease-specific factors, which limits identification of potentially more modifiable drivers of disparities; and (3) little to no research directly compares Hispanic and Black groups, which could help guide intervention approaches.

We designed this study to more fully describe minority YA with type 1 diabetes and identify factors other than SES that account for disparity in glycemic control. We used a targeted, inclusive approach to recruit a diverse and economically vulnerable national population of YA, equally representing Black, Hispanic, and White racial-ethnic groups. We collected an array of patient-reported and chart-extracted variables related to SES, SDOH, and disease-specific variables to capture relevant risk factors. The primary objectives of this study were to (1) describe and compare SES, SDOH, diabetes management factors, and HbA1c levels among Black, Hispanic, and White YA and 2) determine what more modifiable factors may be driving disparities besides SES. Because minorities and YA are very difficult to recruit in research studies, utilization of one-time data capture provided a unique and in-depth view of social determinants of health and inequity that is not otherwise possible in this depth.

## Materials and Methods

### Study design and participants

The Young Adult Racial Disparities in Type 1 Diabetes study involved one-time cross-sectional data collection. This design was chosen to facilitate recruitment and data capture of minority YA with type 1 diabetes, who are severely underrepresented in clinical research studies (22).

Six Type 1 Diabetes Exchange clinic network sites in different urban geographical regions across the United States were selected to participate based on clinic estimates of YA and minority (Black and Hispanic) type 1 diabetes patients. The sites included: (1) Pediatric centers: Children’s Hospital of Los Angeles (Los Angeles, CA), University of Florida (Gainesville, FL), and Baylor Texas Children’s Hospital (Houston, TX); (2) Adult centers: Henry Ford Health System (Detroit, MI); and (3) Combined pediatric-adult centers: Children’s Hospital of Philadelphia-University of Pennsylvania (Philadelphia, PA) and Barbara Davis Center for Diabetes (Aurora, CO). Sites enrolled all participants outside of their existing Type 1 Diabetes Exchange registries to promote inclusion of minority YA who may not usually participate in research. Additional site information is available in the supplement (23).

Participants were included if they were 18 to 25 years old and had a clinical diagnosis of type 1 diabetes for at least 6 months, English-reading ability, and presence of at least 1 HbA1c value in the past 12 months. Participants were excluded if they were taking glucocorticoids, were currently pregnant, had a developmental disability that would preclude procedural requirements of independently filling out survey measures, or were of a self-reported race-ethnicity other than the prespecified racial-ethnic groups.

### Procedures

Recruitment and enrollment occurred between November 2017 and October 2018. A sample size of 300 young adults was calculated based on power calculations to detect at least 0.5% difference in HbA1c levels. This sample size also enabled adequate power (>90%) to detect meaningful differences in most of the measures. We used recruitment targets to ensure equal representation of White, Black, and Hispanic participants, and made efforts to recruit participants who did not show to regularly scheduled visits. The Type 1 Diabetes Exchange acted as the coordinating center for all study-related matters. Each site individually recruited, consented, and enrolled participants with their own study staff. Written informed consent was obtained before collecting any study-specific information. We maintained regular meetings between Type 1 Diabetes Exchange study staff, site study staff, site principal investigators, and the main principal investigator to ensure consistent procedures across sites. The coordinators were instructed periodically to maintain enrollment logs and submit completed logs at study completion. This study was approved by a central institutional review board at the Jaeb Center for Health Research.

After consent and enrollment, participants completed questionnaires through REDCap on a tablet or computer. All language in the consent and survey was in English and at or below a ninth-grade reading level. The survey took 15 to 40 minutes to complete, and participants were compensated for their time. Given the sensitive and private nature of certain questions, YA participants completed the survey in a private room without other family members and could opt out of any questions. Site study staff extracted additional data from participant medical charts. All study data were managed and stored by a central team at the Type 1 Diabetes Exchange.

### Measurements

This study evaluated an extensive list of potential SES, SDOH, and disease-specific variables that might explain observed racial-ethnic disparities in glycemic control in YA with type 1 diabetes. We organized variables in the following domains: (1) SES, (2) treatment regimen, (3) care setting, (4) psychosocial factors, and (5) self-management. Measures are included elsewhere (23).

### Main measures

#### Race-ethnicity.

Race-ethnicity was self-reported using the U.S. Census 2000 questionnaire. We only included Black and White races in this study because our a priori hypotheses were based on prior literature examining these groups. We also could not ensure sufficient recruitment of other races at the selected sites to ensure adequate representation. For ethnicity, most Hispanic participants purposely declined to identify their race. This trend is in line with other studies (24, 25). Therefore, participants designated themselves as non-Hispanic White, non-Hispanic Black, or Hispanic.

#### Glycemic control.

Study staff provided all HbA1c values recorded for each participant within the past 12 months (both point-of-care HbA1c and laboratory tests). For these analyses, we report and use the mean recorded HbA1c value for each participant and calculate disparity as the difference in mean HbA1c levels between White and Black, White and Hispanic, and Black and Hispanic participants.

### Other variables of interest

#### Socioeconomic status.

We used multiple patient-reported and chart-extracted measurements to maximize our ability to accurately capture SES. Measures included insurance type, highest education attained, household income, neighborhood poverty, social status, and food insecurity. We extracted insurance information from the medical chart and categorized it at the site level into a binary variable: private or public/other/none. We asked participants to report their highest education level and annual household income. We assessed neighborhood poverty level by using 5-digit ZIP codes and available census tract data from the U.S. Census (26). We assessed social status by using the Hollingshead Index, which is a self-reported measure containing 15 questions that evaluates education and job type for each gainfully employed person in the household (27). Last, we used the short form of the U.S. Department of Agriculture Food Security Scale to measure household food security (28).

#### Treatment regimen and care setting.

We extracted information from participant medical charts on current insulin pump use and continuous glucose monitor (CGM) use in the past. We categorized care settings into 3 groups: pediatric, adult, or combined pediatric-adult diabetes centers.

#### Psychosocial factors.

Participants completed the Adverse Childhood Experiences (ACE) questionnaire to elicit information on childhood abuse, neglect, and family/household dysfunction, among other experiences (29). We used ACE as a categorical variable given prior work reflecting a dose-response relationship between 0, 1 and 4, and > 4 ACEs and diabetes incidence/control (30). All categories ≥ 1 were collapsed given few participants with scores > 1. Participants also completed the Diabetes Distress Scale, which is an assessment of diabetes-related emotional stress and is associated with glycemic control (31).

#### Disease self-management.

Participants completed several measures of disease self-management: (1) the Diabetes Self-care Inventory (revised), a self-report measure of perceived adherence to diabetes self-care recommendations (32); (2) the Diabetes Numeracy Test 5, which contains 5 math problems for type 1 diabetes self-management (33); and (3) the Single Item Literacy Screener, which measures health literacy (34).

### Data analysis

SES, treatment regimen, care setting, psychosocial factors, and self-management variables were compared between each pair of race-ethnicity groups (Black, Hispanic, White) using a Fisher’s exact test or Wilcoxon rank-sum test, as appropriate. These comparisons used available cases only. Missing data were minimal for most variables and is reported in the tables.

HbA1c was compared between race-ethnicity groups in a linear regression model adjusted for age, sex, and diabetes duration. Because HbA1c was somewhat right skewed, the model was fit with robust linear regression using MM estimation. Robust regression was chosen instead of a transformation because it was important to be able to interpret the regression coefficients on the original scale.

To examine how measures of SES, treatment regimen, care setting, psychosocial factors, and self-management might account for any observed racial-ethnic disparity in HbA1c, we first added each measure individually to the regression model. To examine the effect of the measures when considered together, we sequentially added them to the model in groups in the following order: SES, treatment regimen, care setting, psychosocial variables, and self-management. Variables were added in groups rather than 1 at a time because it was expected that many of them would be highly correlated with each other, so it would be impossible to accurately estimate the individual contribution. The order of variables was chosen to assess the contribution of other factors to disparity after adjustment for well-known structural risk factors, such as SES. Missing data were handled using Rubin’s multiple imputation so that all participants were included in every model.

We adjusted 2-sided *P* values and 95% confidence intervals for multiple comparisons in all analyses to control for the false discovery rate using the adaptive Benjamini-Hochberg procedure. We used SAS software, version 9.4 (SAS Institute Inc., Cary, NC), for analyses.

## Results

### Participant characteristics

Three hundred diverse YA with type 1 diabetes participated in the study per recruitment targets: 33% White (n = 100), 32% Black (n = 97), and 34% Hispanic (n = 103) (Table 1). Participants exhibited a wide range of socioeconomic indicators, with one-half on public insurance or without insurance, and more than one-half reporting annual household incomes of less than $50,000 (Table 1). Mean HbA1c for the sample was 9.0% (75 mmol/mol). Fewer than one-half of participants were using an insulin pump at the time of the survey (43%, n = 129) or had ever used a CGM (45%, n = 135). There was a roughly even distribution of participants from pediatric, adult, and combined pediatric-adult care settings (Table 1).

**Table 1.** Participant Characteristics

	Overall N = 300	NH WhiteN = 100	NH BlackN = 97	Hispanic N = 103	NH White vs. NH Black	NH White vs. Hispanic	Hispanic vs. NH Black
Demographic variables							
Age (yrs) – median (Q1, Q3)	20 (19, 22)	21 (20, 23)	21 (19, 22)	20 (19, 20)			
Female	166 (55%)	59 (59%)	54 (56%)	53 (51%)			
Diabetes duration (yrs) – median (Q1, Q3)	10 (7, 14)	12 (9, 16)	10 (7, 15)	10 (6, 13)			
Socioeconomic status							
Insurance (public/none)	155 (52%)	22 (22%)	55 (57%)	78 (76%)	<0.001	<0.001	0.018
High school education or less	29 (10%)	24 (24%)	64 (66%)	72 (70%)	<0.001	<0.001	0.64
Annual household income <$50 000	171 (61%)	31 (33%)	70 (74%)	70 (74%)	<0.001	<0.001	0.69
% Poverty in census tract – median (Q1, Q3)	16 (9, 25)	9 (6, 18)	22 (14, 33)	19 (12, 27)	<0.001	<0.001	0.042
Hollingshead Index – median (Q1, Q3)	43 (32, 53)	50 (38, 58)	41 (31, 51)	35 (24, 47)	<0.001	<0.001	0.040
Food insecurity	66 (22%)	14 (14%)	28 (29%)	24 (23%)	0.014	0.19	0.42
Treatment regimen							
Insulin pump	129 (43%)	72 (72%)	17 (18%)	40 (39%)	<0.001	<0.001	0.011
CGM ever	135 (45%)	70 (71%)	27 (28%)	38 (37%)	<0.001	<0.001	0.18
Care setting							
Pediatric	111 (37%)	25 (25%)	15 (15%)	71 (69%)			
Adult	67 (22%)	31 (31%)	34 (35%)	2 (2%)			
Combined	122 (41%)	44 (44%)	48 (49%)	30 (29%)			
Psychosocial factors							
≥ 1 adverse childhood experience	173 (58%)	47 (47%)	69 (71%)	57 (55%)	<0.001	0.36	
D0.040iabetes distress –median (Q1, Q3)	1.8 (1.3, 2.7)	1.7 (1.2, 2.5)	2.0 (1.5, 3.0)	1.7 (1.3, 2.5)	0.023	0.89	0.07
Self-management							
Diabetes Self-care Inventory – mean (SD)	3.4 (0.7)	3.5 (0.5)	3.2 (0.8)	3.5 (0.7)	0.006	0.84	0.040
Diabetes numeracy – median (Q1, Q3)	4 (3, 5)	5 (4, 5)	4 (2, 5)	4 (2, 5)	<0.001	<0.001	0.74
Health nonliterate	40 (13%)	7 (7%)	11 (11%)	22 (21%)	0.33	0.028	0.06

Abbreviations: CGM, continuous glucose monitor; NH, non-Hispanic; Q, quarter.

Mean and standard deviation were reported for variables with an approximately normal distribution and the median, 25th, and 75th percentiles were reported for variables with skewed distributions.

*P* values are calculated from a Wilcoxon rank-sum test or a Fisher’s exact test, as appropriate. *P* values have been adjusted for multiple comparisons to control the false discovery rate.

Missing data: income 18 (6%), % poverty in Census Tract 6 (2%), Hollingshead Index 43 (14%), CGM use ever 1 (<1%), diabetes self-care 1 (<1%), hospitalizations 2 (<1%). All other variables have no missing data.

### Racial-ethnic comparisons

Mean ± SD HbA1c for Black YA was 10.7 ± 2.4% (93 ± 26 mmol/mol), for Hispanic YA was 9.3 ± 2.2% (78 ± 24 mmol/mol), and for White YA was 8.5 ± 1.8% (69 ± 20 mmol/mol). Adjusting for age, sex, and diabetes duration, Black participants had HbA1c levels 2.26 percentage points (24.7 mmol/mol) higher than White participants (*P* < 0.001) (Tables 2 and 3). Hispanic participants had HbA1c 0.64 percentage points (7.0 mmol/mol) higher than White participants, but this was not statistically significant (*P* = 0.15).

**Table 2.** Variable Effect on Racial-Ethnic Disparity in HbA1c (%)

	NH Black vs. NH White		Hispanic vs. NH White	
Predictor	β (95% CI)^*a*^	*P* Value^*a*^	β (95% CI)^*a*^	*P* Value^*a*^
**Race**-**ethnicity only**	2.26 (1.46-3.07)	<0.001	0.64 (-0.14 to 1.43)	0.15
**Socioeconomic status**				
** **Health insurance	2.13 (1.46-2.81)	** < 0.001**	0.48 (-0.30 to 1.26)	0.29
** **Highest education attained^*b*^	1.81 (1.16-2.47)	** < 0.001**	0.34 (-0.35 to 1.04)	0.41
** **Annual household income^*b*^	1.93 (1.24-2.63)	** < 0.001**	0.34 (-0.40 to 1.08)	0.45
** **% poverty in Census Tract	1.98 (1.30-2.67)	** < 0.001**	0.45 (-0.28 to 1.19)	0.29
** **Hollingshead Index	2.00 (1.34-2.65)	** < 0.001**	0.25 (-0.48 to 0.98)	0.59
** **Food insecurity	2.18 (1.50-2.86)	** < 0.001**	0.62 (-0.15 to 1.38)	0.15
**Treatment regimen**				
** **Insulin pump	1.76 (1.06-2.46)	** < 0.001**	0.41 (-0.31 to 1.13)	0.33
** **CGM use ever	2.10 (1.42-2.78)	** < 0.001**	0.53 (-0.22 to 1.28)	0.21
**Care Setting**				
** **Pediatric vs. adult/combined	2.16 (1.51-2.82)	** < 0.001**	0.92 (0.00-1.84)	0.05
**Psychosocial factors**				
** **Adverse childhood experiences	2.22 (1.53-2.90)	** < 0.001**	0.63 (-0.15 to 1.41)	0.15
** **Diabetes distress	1.97 (1.37-2.56)	** < 0.001**	0.58 (-0.13 to 1.29)	0.15
**Self-management**				
** **Diabetes Self-care Inventory	1.89 (1.29-2.49)	** < 0.001**	0.56 (-0.14 to 1.26)	0.15
** **Diabetes numeracy	2.05 (1.38-2.71)	** < 0.001**	0.46 (-0.27 to 1.20)	0.28
** **Health nonliterate	2.27 (1.43-3.11)	** < 0.001**	0.66 (-0.14 to 1.46)	0.15

Abbreviations: CI, confidence interval; NH, non-Hispanic.

^*a*^Each variable was added to a linear regression model adjusting for race/ethnicity, age, sex, and diabetes duration. *P* values and CI have been adjusted for multiple comparisons to control the false discovery rate.

^*b*^Education and income are considered ordinal.

**Table 3.** Multivariate Model of Race-Ethnicity and HbA1c (%)

	Model 1 (Race-Ethnicity)^*a*^		Model 2 (Model 1 + SES) ^*a*^		Model 3 (Model 2 + Diabetes Technology)^*a*^		Model 4 (Model 3 + Site Type)^*a*^		Model 5 (Model 4 + Psychosocial)^*a*^		Final Model (Model 5 + Self-management)^*a*^	
	β (95% CI)	*P* Value	β (95% CI)	*P* Value	β (95% CI)	*P* Value	β (95% CI)	*P* Value	β (95% CI)	*P* Value	β (95% CI)	*P* Value
**Race-ethnicity**												
NH Black vs. NH White	2.26 (1.46-3.07)	<0.001	1.41 (0.69-2.13)	**<0.001**	1.04 (0.29-1.79)	**0.007**	0.90 (0.14-1.65)	**0.019**	0.81 (0.10-1.52)	**0.025**	0.70 (0.02-1.38)	**0.044**
Hispanic vs. NH White	0.64 (-0.14 to 1.43)	0.15	-0.04 (-0.78 to 0.69)	0.91	-0.17 (-0.92 to 0.58)	0.73	0.10 (-0.64 to 0.84)	0.79	0.08 (-0.62 to 0.78)	0.82	0.02 (-0.65 to 0.70)	0.94
**Socioeconomic status**												
Insurance (private vs. public/none)	-	-	0.03 (-0.57 to 0.62)	0.93	0.19 (-0.42 to 0.80)	0.55	0.19 (-0.41 to 0.80)	0.53	0.03 (-0.54 to 0.60)	0.92	0.30 (-0.25 to 0.84)	0.28
Highest attained education^*b*^	-	-	-0.62 (-1.12 to 0.13)	**0.006**	-0.59 (-1.08 to 0.11)	**0.008**	-0.61 (-1.10 to 0.12)	**0.006**	-0.66 (-1.15 to 0.17)	**0.002**	-0.55 (-1.02 to 0.08)	**0.014**
Annual household income^*b*^	-	-	-0.12 (-0.44 to 0.19)	0.44	-0.13 (-0.44 to 0.18)	0.40	-0.19 (-0.50 to 0.12)	0.22	-0.19 (-0.49 to 0.11)	0.21	-0.23 (-0.54 to 0.07)	0.16
% Neighborhood poverty	-	-	0.02 (-0.01 to 0.04)	0.19	0.02 (-0.01 to 0.04)	0.18	0.02 (-0.01 to 0.04)	0.16	0.01 (-0.01 to 0.03)	0.28	0.01 (-0.01 to 0.04)	0.20
Hollingshead Index	-	-	-0.02 (-0.04 to 0.01)	0.18	-0.01 (-0.04 to 0.01)	0.22	-0.01 (-0.03 to 0.01)	0.34	-0.01 (-0.03 to 0.01)	0.57	-0.01 (-0.03 to 0.01)	0.41
Food insecurity	-	-	0.48 (-0.19 to 1.15)	0.18	0.42 (-0.23 to 1.07)	0.21	0.41 (-0.22 to 1.05)	0.21	0.08 (-0.53 to 0.70)	0.79	0.23 (-0.35 to 0.81)	0.43
**Treatment regimen**												
Insulin regimen (pump vs. injections)	-	-	-	-	-0.80 (-1.53 to -0.08)	0.023	-0.72 (-1.40 to -0.04)	0.035	-0.63 (-1.25 to -0.01)	0.045	-0.44 (-1.02 to 0.14)	0.16
CGM user ever	-	-	-	-	-0.11 (-0.68 to 0.46)	0.71	-0.19 (-0.76 to 0.38)	0.51	-0.11 (-0.65 to 0.43)	0.68	-0.18 (-0.70 to 0.33)	0.49
**Care setting**												
Pediatrics vs. adult/combined	-	-	-	-	-	-	-0.82 (-1.57 to -0.08)	0.025	-0.71 (-1.38 to -0.04)	0.036	-0.71 (-1.37 to -0.05)	0.031
**Psychosocial factors**												
Adverse childhood experiences	-	-	-	-	-	-	-	-	-0.37 (-0.90 to 0.15)	0.18	-0.38 (-0.90 to 0.13)	0.17
Diabetes distress	-	-	-	-	-	-	-	-	0.62 (0.27-0.97)	<0.001	0.20 (-0.08 to 0.47)	0.18
**Self-management**												
Self-care inventory	-	-	-	-	-	-	-	-	-	-	-0.99 (-1.54 to -0.44)	<0.001
Diabetes numeracy	-	-	-	-	-	-	-	-	-	-	-0.16 (-0.35 to 0.02)	0.10
Health nonliterate	-	-	-	-	-	-	-	-	-	-	-0.49 (-1.21 to 0.23)	0.20

Abbreviations: CI, confidence interval; NH, non-Hispanic; SES, socioeconomic status.

^*a*^Each group of variables was added sequentially to a model of HbA1c adjusting for race/ethnicity, age, sex, and diabetes duration. *P* values and CI have been adjusted for multiple comparisons to control the false discovery rate.

^*b*^Education and income are considered ordinal.

Compared with White participants, both Black and Hispanic participants had significantly worse socioeconomic indicators with lower annual household income, lower attained education, lower social status, and higher neighborhood poverty (Table 1). In addition, far fewer minority participants were on insulin pump treatment (18% Black, 39% Hispanic, vs 72% White, *P* < 0.001) or had ever used a CGM (28% Black, 37% Hispanic, vs 71% White, *P* < 0.001) (Table 1).

Compared with White and Hispanic participants, Black YA reported the higher neighborhood poverty levels, lower social status, higher food insecurity, more adverse childhood experiences, higher levels of diabetes distress, and lower diabetes self-management scores (*P* < 0.001 for all) (Table 1). In addition, Black YA had the lowest insulin pump use, despite similar rates of public insurance as Hispanic YA (Table 1).

### Explanation of racial-ethnic disparity in glycemic control

**Figure 1.**
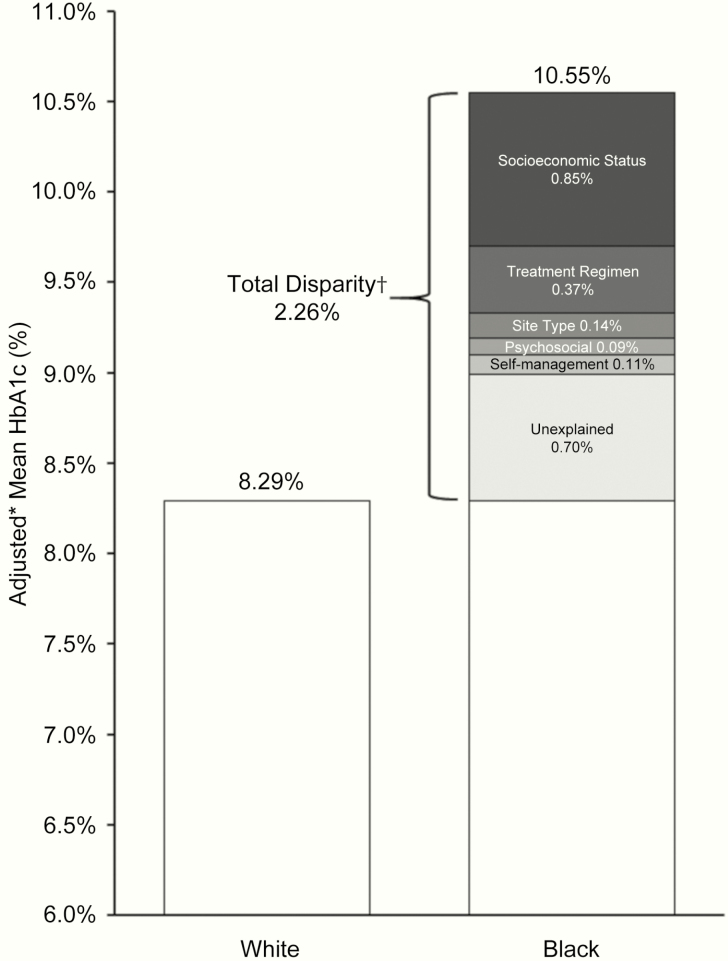
Portion of the total HbA1c disparity between Black and White participants that is explained by each variable group.

When examining each variable’s effect on glycemic control after adjusting for age, sex, and diabetes duration (Table 2), all variables were associated with glycemic control in Black but not Hispanic YA. In multivariate sequential modeling after controlling for SES factors (model 2, Table 3), Hispanic YA appeared more like White participants in terms of glycemic control. Meanwhile, Black YA continued to have stark disparity in HbA1c levels, with diabetes technology use, care setting, diabetes distress, and self-care exerting the greatest influence on Black–White glycemic disparity (Table 3, Fig. 1).

## Discussion

We performed this study in a diverse national population of YA with type 1 diabetes to describe disparities, examine the effect of variables other than SES on glycemic control, and compare differences between minority groups. Black and Hispanic YA exhibited lower SES, much lower diabetes technology use, and higher HbA1c levels than White YA. Across the board, Black YA had the lowest scores on all variables, most significantly for self-reported psychosocial and self-management factors. After accounting for SES, Hispanic YA were similar to White YA exhibiting no difference in glycemic control, whereas Black YA continued to have much higher HbA1c levels than White YA. The variables that contributed most to the remaining Black–White glycemic disparity after SES were diabetes technology use, diabetes distress, and diabetes self-management. Our findings indicate that increased effort to encourage and facilitate insulin pump and CGM use among Black and Hispanic YA may be one avenue to mitigate racial-ethnic inequity. Our results also demonstrate that clinicians may be able to improve HbA1c levels among Hispanic YA by directly addressing social needs, whereas Black YA may need further support with tailored reeducation and behavioral interventions that target diabetes distress and self-care.

To our knowledge, this study is the first in YA designed to test how multiple patient-reported and chart-extracted variables account for racial-ethnic disparity in glycemic control. We used targeted recruitment to enable equal representation and comparison of Black and Hispanic groups. Our results demonstrated that SDOH composed the largest portion of glycemic disparity; however, we were also able to identify clear modifiable factors that account for remaining inequity, such as diabetes technology use, diabetes distress, and self-management. We also demonstrated key differences between Black and Hispanic YA, which may help target and tailor appropriate interventions. An unexplained disparity of 0.7% in HbA1c levels remained after accounting for all variables, most of which can be explained by biologic differences in hemoglobin glycation between Black and White populations, accounting for approximately 0.5% in HbA1c (18).

Although it was expected that Black and Hispanic YA had significant disparity in economic variables compared with White YA, the degree to which both groups had lower diabetes technology use was surprising. This is especially poignant given that participants were cared for in subspecialized diabetes centers and resided in states with generous insurance plans that covered diabetes technology. Our results demonstrate that the impact of diabetes technology use on glycemic control was large, even after adjustment for SES, rivaling treatment effects of new diabetes medications (35). Although prior research has highlighted racial-ethnic disparities in diabetes technology among youth with type 1 diabetes (36, 37), our results show that racial-ethnic disparities exist despite being covered for these devices. In addition, we show that Black YA had lower diabetes technology use than Hispanic YA, despite similar public insurance coverage rates and SES levels. Thus, cost may not be the only driver of racial-ethnic disparities in technology use. That more Hispanic patients were in pediatric care could have accounted for their slightly better rates of technology use over Blacks, but it does not fully explain why both minority groups had much lower use than White YA.

Implicit bias in prescribing practices must be considered. Studies evaluating prescription of statins, psychotropic medications, and opioids for pain control suggest that prescribing practices create racial-ethnic disparities (38-40). Whether societal issues for Black YA related to racism, mistrust of health care systems, and fractured support networks (41, 42) influence provider prescription practices of diabetes technology are unknown. Further, it is unknown whether cultural norms influence patient acceptance of advanced diabetes technologies. Studies highlighting literacy barriers in technology promotional materials and minority patient preferences for quality of life over medical outcomes underscores the need to more fully explore patient factors in acceptance of diabetes technology (43, 44). As emerging technologies, such as closed loop systems, become more readily available in the near future and have clear glycemic advantage over existing treatment regimens (45, 46), more research is needed to examine minority preferences and challenges as well as patient–provider interactions surrounding technology. Moreover, more lenient eligibility criteria for use of these advanced systems and invention of more affordable technology may be necessary to prevent widening disparities (47).

We also found that Black–White disparity in glycemic control stemmed from higher levels of self-reported diabetes distress and lower self-management scores in Black YA. The exact causes of these self-reports cannot be discerned from this study, but prior studies have suggested that low social and disease-related support in low SES communities contributes to higher perceived stress and lower self-management (48). In the YA context, support for diabetes-related distress and self-management may be particularly necessary during the developmental transition to adulthood as well (10). In our study, Black YA reported lower social status and higher neighborhood poverty levels than White or Hispanic YA. Whether these neighborhood and societal indicators are markers for lower overall social and disease-related support needs to be further examined.

It is notable that Hispanic YA reported equivalent diabetes distress and self-management to White YA, despite similar economic insecurity to Black YA. Hispanic participants in our study were more likely to be in pediatric care than Black participants, which may have provided them access to better safety nets. However, several studies have suggested that Hispanic populations are shielded from some of the negative effects of SES through the “barrio effect,” which involves residence in neighborhoods with shared characteristics of intact family structures, community institutions, and kinship networks (49-51). These cultural factors may be protective among Hispanic individuals and communities (50) and may have contributed to the observed differences between Hispanic and Black YA in our study. Given our results, clinicians should consider evaluating and strengthening social support among vulnerable YA, whether through family, neighborhood, peer, virtual, or other community networks. In fact, a wealth of literature underscores how resilience can be protective against the deleterious effects of SES, specifically in youth with type 1 diabetes (52-54). Pediatric care may be a favorable model to promote resilience for YA. However, even in resource-strained settings such as adult care, engaging the multifaceted networks of YA to support strengths and promote resilience may be a unique method of mitigating racial-ethnic inequity in outcomes.

This study has several limitations. First, it is cross-sectional, thus limiting our ability to make causal inferences. We specifically chose this design to maximize recruitment and data capture, offering insight into a severely underrepresented population with the hope that any data would be value added. Second, although this is the largest study of social contextual factors and glycemic control in minority YA with type 1 diabetes to date, there may have been limited power to detect potentially important differences and moderating or mediating relationships. Third, we recruited participants from specialty diabetes centers and used English-only materials, which could have skewed the population toward a more educated, engaged, and acculturated Hispanic population. Fourth, we found that more Hispanic participants were from pediatric centers than Black participants, which may have selected for a Hispanic population that had better support because of the site of care. Nevertheless, our results showing more favorable psychosocial and outcome variables in the Hispanic group mirror other larger studies of other diseases that demonstrate protective cultural aspects (55).

This study focused on a population of high relevance and used a new approach to identify modifiable drivers of disparities in a national population of YA with type 1 diabetes. We uncovered meaningful racial-ethnic disparities in glycemic control, SDOH, and diabetes-specific factors. Our results highlight several critical priority areas to decrease racial-ethnic inequity among minority YA. These include a need to increase diabetes technology use, provide clinicians with training to address social needs with connection to existing social programs, and tailor support for diabetes self-management and diabetes distress to which leverages existing YA social networks. Overall, clinicians need to adapt current care approaches to caring for minority YA with type 1 diabetes that takes into account developmental, cultural, historical, and environmental contexts. These changes have the potential to prevent inequity from enduring into later adulthood.

## Abbreviations

ACE: Adverse Childhood Experiences
CGM: continuous glucose monitoring
SDOH: Social Determinants of Health
SES: socioeconomic status
YA: young adult

## Additional Information

***Disclosure Summary*:** None of the authors have relevant disclosures to this manuscript.

***Data Availability:*** The datasets generated during and/or analyzed during the current study are not publicly available but are available from the corresponding author on reasonable request.

## References

[1] Dabelea D, Mayer-Davis EJ, Saydah S, et al. Prevalence of type 1 and type 2 diabetes among children and adolescents from 2001 to 2009. JAMA. 2014;311(17):1778-1786. 10.1001/jama.2014.3201 24794371 PMC4368900

[2] Dabelea D, Stafford JM, Mayer-Davis EJ, et al. Association of type 1 diabetes vs type 2 diabetes diagnosed during childhood and adolescence with complications during teenage years and young adulthood. JAMA. 2017;317(8):825-835. 10.1001/JAMA.2017.0686 28245334 PMC5483855

[3] Centers for Disease Control and Prevention. National Diabetes Statistics Report, 2020. Accessed May 19, 2020.

[4] Clements MA, Foster NC, Maahs DM, et al.; T1D Exchange Clinic Network Hemoglobin A1c (HbA1c) changes over time among adolescent and young adult participants in the T1D exchange clinic registry. Pediatr Diabetes. 2016;17(5):327-336. 26153338 10.1111/pedi.12295

[5] Schwandt A, Hermann JM, Rosenbauer J, et al.; DPV Initiative Longitudinal trajectories of metabolic control from childhood to young adulthood in type 1 diabetes from a large German/Austrian registry: a group-based modeling approach. Diabetes Care. 2017;40(3):309-316. 28007778 10.2337/dc16-1625

[6] Kahkoska AR, Shay CM, Crandell J, et al. Association of race and ethnicity with glycemic control and hemoglobin A 1c levels in youth with type 1 diabetes. JAMA Netw Open. 2018;1(5):e181851 10.1001/jamanetworkopen.2018.1851 30370425 PMC6203341

[7] Miller KM, Foster NC, Beck RW, et al.; T1D Exchange Clinic Network Current state of type 1 diabetes treatment in the U.S.: updated data from the T1D Exchange clinic registry. Diabetes Care. 2015;38(6):971-978. 25998289 10.2337/dc15-0078

[8] Livingstone SJ, Levin D, Looker HC, et al. Estimated life expectancy in a Scottish cohort with type 1 diabetes, 2008–2010. JAMA. 2015;313(1):37-44. 10.1001/jama.2014.16425 25562264 PMC4426486

[9] Rhodes ET, Prosser LA, Hoerger TJ, Lieu T, Ludwig DS, Laffel LM Estimated morbidity and mortality in adolescents and young adults diagnosed with type 2 diabetes mellitus. Diabet Med. 2012;29(4):453-463. 22150528 10.1111/j.1464-5491.2011.03542.x

[10] Arnett JJ Emerging adulthood. A theory of development from the late teens through the twenties. Am Psychol. 2000;55(5):469-480. 10842426

[11] Crossen SS, Wilson DM, Saynina O, Sanders LM Outpatient care preceding hospitalization for diabetic ketoacidosis. Pediatrics. 2016;137(6):Epub ahead of print. 10.1542/peds.2015-3497 PMC4894257 27207491

[12] Kipps S, Bahu T, Ong K, et al. Current methods of transfer of young people with Type 1 diabetes to adult services. Diabet Med. 2002;19(8):649-654. 12147145 10.1046/j.1464-5491.2002.00757.x

[13] Bryden KS, Dunger DB, Mayou RA, Peveler RC, Neil HA Poor prognosis of young adults with type 1 diabetes: a longitudinal study. Diabetes Care. 2003;26(4):1052-1057. 12663572 10.2337/diacare.26.4.1052

[14] Bryden KS, Peveler RC, Stein A, Neil A, Mayou RA, Dunger DB Clinical and psychological course of diabetes from adolescence to young adulthood: a longitudinal cohort study. Diabetes Care. 2001;24(9):1536-1540. 11522695 10.2337/diacare.24.9.1536

[15] Garvey KC, Telo GH, Needleman JS, Forbes P, Finkelstein JA, Laffel LM Health care transition in young adults with type 1 diabetes: perspectives of adult endocrinologists in the U.S. Diabetes Care. 2016;39(2):190-197. 26681724 10.2337/dc15-1775 PMC4722944

[16] Garvey KC, Foster NC, Agarwal S, et al. Health care transition preparation and experiences in a U.S. national sample of young adults with type 1 diabetes. Diabetes Care. 2017;40(3):317-324. 28007779 10.2337/dc16-1729 PMC5319474

[17] Lotstein DS, Seid M, Klingensmith G, et al.; SEARCH for Diabetes in Youth Study Group Transition from pediatric to adult care for youth diagnosed with type 1 diabetes in adolescence. Pediatrics. 2013;131(4):e1062-e1070. 23530167 10.1542/peds.2012-1450 PMC4535025

[18] Bergenstal RM, Gal RL, Connor CG, et al; M P for the TERDSG Racial differences in the relationship of glucose concentrations and hemoglobin A1c Levels. Ann Intern Med. 2017;167(2):95-102. 28605777 10.7326/M16-2596

[19] Commission on Social Determinants of Health. Closing the gap in a generation: Health equity through action on the social determinants of health. Geneva: World Health Organization, 2008. who.int/social_determinants/thecommission/finalreport. Accessed May 19, 2020. 10.1016/S0140-6736(08)61690-6 18994664

[20] Centers for D isease Control and Prevention. Social Determinants of Health: Know What Affects Health, 2020. cdc.gov/socialdeterminants/index.htm. Accessed May 19, 2020.

[21] World Health Organization. Healthy People 2020: Social Determinants of Health https://www.healthypeople.gov/2020/topics-objectives/topic/social-determinants-of-health. Accessed April 1, 2018.

[22] Agarwal S, Hilliard M, Butler A Disparities in care delivery and outcomes in young adults with diabetes. Curr Diab Rep. 2018;18(9):65. 30008025 10.1007/s11892-018-1037-x

[23] Agarwal S, et al. Supplemental tables for “Racial-Ethnic Inequity in Young Adults With Type 1 Diabetes.” Dryad. 2020 10.5061/dryad.2280gb5p3. PMC7457963 32382736

[24] Hirschman C, Alba R, Farley R The meaning and measurement of race in the U.S. census: glimpses into the future. Demography. 2000;37(3):381-393. 10953811

[25] Center PR Seeking better data on Hispanics, Census Bureau may change how it asks about race https://www.pewresearch.org/fact-tank/2017/04/20/seeking-better-data-on-hispanics-census-bureau-may-change-how-it-asks-about-race/. Published 2017 Accessed March 4, 2020.

[26] The United States Census Bureau. The United States Census Bureau: Race https://www.census.gov/topics/population/race/about.html. Accessed April 7, 2018.

[27] Hollingshead AA. Four-Factor Index of Social Status. New Haven, CT: Yale University Department of Psychology, 1975.

[28] Blumberg SJ, Bialostosky K, Hamilton WL, Briefel RR The effectiveness of a short form of the Household Food Security Scale. Am J Public Health. 1999;89(8):1231-1234. 10432912 10.2105/ajph.89.8.1231 PMC1508674

[29] Felitti VJ, Anda RF, Nordenberg D, et al. Relationship of childhood abuse and household dysfunction to many of the leading causes of death in adults. Am J Prev Med. 1998;14(4):245-258. 9635069 10.1016/s0749-3797(98)00017-8

[30] Huffhines L, Noser A, Patton SR The link between adverse childhood experiences and diabetes. Curr Diab Rep. 2016;16(6):54. 27112958 10.1007/s11892-016-0740-8 PMC5292871

[31] Polonsky WH, Anderson BJ, Lohrer PA, et al. Assessment of diabetes-related distress. Diabetes Care. 1995;18(6):754-760. 7555499 10.2337/diacare.18.6.754

[32] Weinger K, Butler HA, Welch GW, La Greca AM Measuring diabetes self-care: a psychometric analysis of the self-care inventory-revised with adults. Diabetes Care. 2005;28(6):1346-1352. 15920050 10.2337/diacare.28.6.1346 PMC1615849

[33] Huizinga MM, Elasy TA, Wallston KA, et al. Development and validation of the Diabetes Numeracy Test (DNT). BMC Health Serv Res. 2008;9(96). 10.1186/1472-6963-8-96 PMC2390531 18452617

[34] Morris NS, MacLean CD, Chew LD, Littenberg B The Single Item Literacy Screener: evaluation of a brief instrument to identify limited reading ability. BMC Fam Pract. 2006;7:21. 16563164 10.1186/1471-2296-7-21 PMC1435902

[35] U.S. Department of Health and Human, Food and Drug Administration, Center for Drug Evaluation and Research (CDER). Guidance for Industry. Diabetes Mellitus: Developing Drugs and Therapeutic Biologics for Treatment and Prevention; 2008 http://www.fda.gov/downloads/Drugs/Guidances/ucm071624.pdf. Accessed February 20, 2020.

[36] Willi SM, Miller KM, DiMeglio LA, et al.; T1D Exchange Clinic Network Racial-ethnic disparities in management and outcomes among children with type 1 diabetes. Pediatrics. 2015;135(3):424-434. 25687140 10.1542/peds.2014-1774 PMC4533245

[37] Clements MA, Schwandt A, Donaghue KC, et al; on behalf of the Australasian Diabetes Data Network (ADDN) Study Group, the T1D Exchange and the GD (DPV) initiative. Five heterogeneous HbA1c trajectories from childhood to adulthood in youth with type 1 diabetes from three different continents – a group‐based modeling approach. Pediatr Diabetes 2019;20(7):920-931. 31418521 10.1111/pedi.12907

[38] Johansen ME, Hefner JL, Foraker RE Antiplatelet and statin use in US patients with coronary artery disease categorized by race/ethnicity and gender, 2003 to 2012. Am J Cardiol. 2015;115(11):1507-1512. 25840577 10.1016/j.amjcard.2015.02.052

[39] Mills AM, Shofer FS, Boulis AK, Holena DN, Abbuhl SB Racial disparity in analgesic treatment for ED patients with abdominal or back pain. Am J Emerg Med. 2011;29(7):752-756. 20825892 10.1016/j.ajem.2010.02.023

[40] Cook BL, Carson NJ, Kafali EN, et al. Examining psychotropic medication use among youth in the U.S. by race/ethnicity and psychological impairment. Gen Hosp Psychiatry. 2017;45:32-39. 28274336 10.1016/j.genhosppsych.2016.12.004 PMC7598773

[41] Brittian AS Understanding African American adolescents’ identity development: a relational developmental systems perspective. J Black Psychol. 2012;38(2):172-200. 23243325 10.1177/0095798411414570 PMC3520439

[42] Karatekin C, Ahluwalia R Effects of adverse childhood experiences, stress, and social support on the health of college students. J Interpers Violence. 2020;35(1-2):150-172. 27920360 10.1177/0886260516681880

[43] Ruelas VF, Walker MA, Peters AL The STEPP-UP Project—designing low literacy teaching tools for use of devices in a minority population. Diabetes. 2018;67(Supplement 1).

[44] Huang ES, Brown SE, Thakur N, et al. Racial/ethnic differences in concerns about current and future medications among patients with type 2 diabetes. Diabetes Care. 2009;32(2):311-316. 19017766 10.2337/dc08-1307 PMC2628700

[45] Brown SA, Kovatchev BP, Raghinaru D, et al.; iDCL Trial Research Group Six-month randomized, multicenter trial of closed-loop control in type 1 diabetes. N Engl J Med. 2019;381(18):1707-1717. 31618560 10.1056/NEJMoa1907863 PMC7076915

[46] Boughton C, Allen JM, Tauschmann M, et al.; CLOuD Consortium Assessing the effect of closed-loop insulin delivery from onset of type 1 diabetes in youth on residual beta-cell function compared to standard insulin therapy (CLOuD study): a randomised parallel study protocol. BMJ Open. 2020;10(3):e033500. 10.1136/bmjopen-2019-033500 PMC7069267 32169925

[47] Lawton J, Kimbell B, Rankin D, et al. Health professionals’ views about who would benefit from using a closed-loop system: a qualitative study. Diabet Med. 2020;1-8. 10.1111/dme.14252 31989684

[48] Barajas CB, Jones SCT, Milam AJ, et al. Coping, discrimination, and physical health conditions among predominantly poor, urban African Americans: implications for community-level health services. J Community Health. 2019;44(5):954-962. 30915675 10.1007/s10900-019-00650-9 PMC6708452

[49] Eschbach K, Ostir GV, Patel KV, Markides KS, Goodwin JS Neighborhood context and mortality among older Mexican Americans: is there a barrio advantage? Am J Public Health. 2004;94(10):1807-1812. 15451754 10.2105/ajph.94.10.1807 PMC1448538

[50] Aranda MP, Ray LA, Snih SA, Ottenbacher KJ, Markides KS The protective effect of neighborhood composition on increasing frailty among older Mexican Americans: a barrio advantage? J Aging Health. 2011;23(7):1189-1217. 21948774 10.1177/0898264311421961 PMC3506387

[51] The Lancet Editorial Board. The Hispanic paradox. Lancet. 2015;385(9981):1918. 10.1016/S0140-6736(15)60945-X 26090624

[52] Hilliard ME, Hagger V, Hendrieckx C, et al. Strengths, risk factors, and resilient outcomes in adolescents with type 1 diabetes: results from diabetes MILES Youth-Australia. Diabetes Care. 2017;40(7):849-855. 28446529 10.2337/dc16-2688 PMC5481988

[53] Hilliard ME, Harris MA, Weissberg-Benchell J Diabetes resilience: a model of risk and protection in type 1 diabetes. Curr Diab Rep. 2012;12(6):739-748. 22956459 10.1007/s11892-012-0314-3

[54] Lukács A, Mayer K, Sasvári P, Barkai L Health-related quality of life of adolescents with type 1 diabetes in the context of resilience. Pediatr Diabetes. 2018;19(8):1481-1486. 30203556 10.1111/pedi.12769

[55] McDonald JA, Paulozzi LJ Parsing the paradox: hispanic mortality in the US by detailed cause of death. J Immigr Minor Health. 2019;21(2):237-245. 29605879 10.1007/s10903-018-0737-2

